# Targeting Oxidative Stress and Inflammation in the Eye: Insights from a New Model of Experimental Autoimmune Uveitis

**DOI:** 10.3390/ijms252312910

**Published:** 2024-11-30

**Authors:** Dmitry V. Chistyakov, Veronika V. Tiulina, Olga S. Gancharova, Viktoriia E. Baksheeva, Sergei V. Goriainov, Natalia G. Shebardina, Vasily A. Ivlev, Sergey V. Komarov, Marina P. Shevelyova, Natalia K. Tikhomirova, Pavel P. Philippov, Vasiliy G. Vasil’ev, Marina G. Sergeeva, Sergei E. Permyakov, Elena N. Iomdina, Philipp O. Tsvetkov, Ivan I. Senin, Evgeni Yu. Zernii

**Affiliations:** 1 Belozersky Institute of Physico-Chemical Biology, Lomonosov Moscow State University, 119992 Moscow, Russia; chistyakof@gmail.com (D.V.C.); tyulina_nika@list.ru (V.V.T.); olgancharova@belozersky.msu.ru (O.S.G.); vbaksheeva@belozersky.msu.ru (V.E.B.); natuskasheb@gmail.com (N.G.S.); tikhomir@belozersky.msu.ru (N.K.T.); pf@belozersky.msu.ru (P.P.P.); mg.sergeeva@gmail.com (M.G.S.); senin@belozersky.msu.ru (I.I.S.); 2 Pharmacy Resource Center, Peoples’ Friendship University of Russia (RUDN University), 117198 Moscow, Russia; goryainovs@list.ru (S.V.G.); chemistron@mail.ru (V.A.I.); vasilyev-vg@rudn.ru (V.G.V.); 3 Skryabin Moscow State Academy of Veterinary Medicine and Biotechnology, 109472 Moscow, Russia; skomarov1977@mail.ru; 4 Institute for Biological Instrumentation, Russian Academy of Sciences, 142292 Pushchino, Russia; marina.shevelyova@gmail.com (M.P.S.); permyakov.s@gmail.com (S.E.P.); 5 Helmholtz National Medical Research Center of Eye Diseases, 105062 Moscow, Russia; iomdina@mail.ru; 6 CNRS, UMR 7051, INP, Inst Neurophysiopathol, Faculté des Sciences Médicales et Paramédicales, Aix Marseille Univ, 13005 Marseille, France

**Keywords:** experimental autoimmune uveitis, recoverin, oxidative stress, aqueous humor lipidomics, oxylipins, polyunsaturated fatty acids, aqueous humor metabolomics, ascorbic acid, mitochondria-targeted antioxidant, SkQ1

## Abstract

Autoimmune uveitis is a relapsing blind-causing ocular condition with complex pathogenesis that is not completely understood. There is a high demand for accurate animal models of experimental autoimmune uveitis (EAU) suitable for elucidating the molecular mechanisms of the disease and testing new therapeutic approaches. Here, we demonstrated that photoreceptor Ca^2+^/Zn^2+^-sensor protein recoverin is a uveoretinal antigen in albino rabbits provoking typical autoimmune chorioretinitis 2–4 weeks after immunization. The pathologic process in recoverin-induced EAU shared features with human disease and included lymphocytic infiltration of the retina, Dalen–Fuchs nodules and foci of subtotal or total retinal atrophy, manifested as a decrease in amplitude of the a-wave of the electroretinogram. In some cases, changes in the retinal vascular pattern and subretinal hemorrhages were also observed. These signs were accompanied by a gradual accumulation of serum antibodies against recoverin. Biochemical examination of the aqueous humor (AH) revealed typical characteristics of inflammation and oxidative stress, including increased levels of TNF-α and IL-6 and decreased levels of IL-10, as well as decreased total antioxidant activity, superoxide dismutase and glutathione peroxidase activities, and increased zinc concentration. Consistently, metabolomic and targeted lipidomic analysis of AH showed high lactate and low ascorbic acid levels in early EAU; increased levels of key pro-inflammatory signaling lipids such as PGE2, TXB2, 11-HETE and Lyso-PAF; and reduced levels of the anti-inflammatory fatty acid DHA in advanced stages of the disease. Uveitic AH became enriched with recoverin, confirming disruption of the blood–ocular barrier and photoreceptor damage. Notably, the application of mitochondria-targeted antioxidant therapy impeded EAU progression by maintaining local antioxidant activity and suppressing TNF-α, IL-6 and PGE2 signaling. Overall, our results demonstrate that recoverin-induced EAU in rabbits represents an accurate model of human autoimmune posterior uveitis and suggest new directions for its therapy that can be trialed using the developed model.

## 1. Introduction

Uveitis can be broadly defined as inflammation of the uvea, the vascularized and pigmented area of the middle layer of the eye that includes the iris, ciliary body and choroid. Uveitis can be infectious or autoimmune and anatomically is classified into anterior, intermediate and posterior forms, as well as panuveitis, depending on which part of the eye is affected. The most common form is anterior uveitis, which manifests as iritis or iridocyclitis, as well as intermediate uveitis characterized by vitritis and peripheral retinal vasculitis. Posterior uveitis (chorioretinitis) is a rarer but more severe condition representing an inflammation of the choroid and retina, the signs of which may include significant vision deterioration, retinal neovascularization, retinal detachment and macular edema [1]. The infectious form of posterior uveitis is challenging, because in addition to eliminating the pathogen, it is necessary to prevent an excessive inflammatory reaction that affects the retina and may lead to irreversible blindness. However, it accounts for only about 20% of posterior uveitis, which is more often of autoimmune origin [2]. Posterior non-infectious uveitis may be associated with systemic autoimmune diseases (Behçet’s disease, Vogt Koyanagi Harada syndrome, Sarcoidosis and Anklyosing spondylitis) or be idiopathic. In particular, autoimmune etiology has Fuch’s heterochromic cyclitis, birdshot chorioretinopathy, multifocal choroiditis, pars planitis and sympathetic ophthalmia [1,3].

Typically, autoimmune uveitis is associated with HLA class I or class II: for example, sympathetic ophthalmia is associated with HLA-DR4, whereas birdshot chorioretinopathy is associated with HLA-A29 [4,5]. Accordingly, the retina contains a high concentration of tissue-specific uveitogenic antigens, primarily the proteins involved in visual transduction [6]. The main driving force of non-infectious uveitis is autoreactive renegade T cells maturing in the thymus. Normally, they are kept at a low level by the action of regulatory T cells, which constantly secrete the immunosuppressive cytokine IL-10. In the case of autoimmune uveitis, however, there is overactivation of autoreactive T cells, which induces severe inflammation [3]. Early studies considered CD4+ Th1 cells (secrete IFN-gamma) and regulatory CD4+ T cells (secrete IL-10 and TGF-beta) to be the main mediators of autoimmune uveitis, but development of this condition in IFN-gamma-deficient mice indicated the involvement of other lymphocyte types and led to the discovery of the key role of Th17 cells [7,8]. Subsequently, the adoptive transfer of Th1 and Th17 cells has been shown to lead to blood–retinal barrier disruption and retinal neovascularization, recruitment of other immune cells and spread of inflammation [9]. The development of autoimmune uveitis is associated with Th cells specific to retinal antigens such as S-antigen/visual arrestin (SAG), interphotoreceptor retinoid-binding protein (IRBP), rhodopsin, phosducin and several other proteins [6]. Indeed, susceptibility to uveitis correlates with a decreased tolerance to these autoantigens: ocular proteins are available for tolerance induction, and their high expression in the thymus correlates with resistance to uveitis and vice versa [10]. The action of serum antibodies against retinal antigens generating during autoimmune uveitis is only apparent after destruction of the blood–retinal barrier but nevertheless significantly exacerbates the disease [8].

The use of retinal antigens, primarily SAG and IRBP, has enabled effective modeling of posterior autoimmune uveitis in several susceptible animal species, including rats, mice, monkeys, guinea pigs and rabbits [11]. Experimental autoimmune uveitis (EAU) is characterized by a proliferation of autoimmune T cells, destruction of the blood–retinal barrier, and infiltration of T cells into the uvea and retina, ultimately leading to visual dysfunction [12]. EAU reproduces well the features of human autoimmune uveitis such as chororoiditis, retinitis, subretinal neovascularization, macular edema and photoreceptor damage [3,13]. Immunization is usually performed in complete Freund’s adjuvant containing inactivated tuberculosis bacteria that activate pattern recognition receptors on innate immune cells. In turn, antigen administration induces clonal expansion of Th1 and Th17, which leads to the development of the inflammatory response (days 14–20) with subsequent regulatory T and B lymphocytes-mediated resolution (days 25–32) [3].

Although lymphocytes from some uveitis patients respond to SAG and IRBP, and these antigens cause EAU in certain animal species (see above), the actual retinal antigens involved human disease have not been identified [3,14]. One of the most probable candidates may be neuronal calcium sensor recoverin, a retinal-specific calcium-binding protein that regulates rhodopsin desensitization by GRK1 and has several other functions in photoreceptor cells [15,16]. Indeed, in early studies, recoverin was recognized as EAU antigen in rats [17,18]. Antibodies against recoverin have been detected in the serum of patients with cancer-associated retinopathy and are thought to penetrate the blood–retinal barrier and inhibit recoverin activity in the visual transduction cascade, causing photoreceptor degeneration [19,20,21]. Subsequently, recoverin was identified as an autoantigen in horses with spontaneous recurrent uveitis [22]. Finally, a more recent study reported that, in patients with non-infectious uveitis, the incidence of antibodies against SAG, IRBP and other retinal antigens (RPE65, TYRP1 and TYRP2) was not significantly different from normal controls, while the incidence of antibodies against recoverin was markedly elevated. Thus, recoverin appears to be the key autoantigen in autoimmune uveitis, especially in birdshot chorioretinopathy [23].

To study autoimmune uveitis and trial therapeutic approaches, there is a high demand of EAU models that most closely replicate the human disease in terms of etiology and pathogenesis at the clinical, functional and structural (cellular) levels. In addition to using the appropriate antigen (i.e., recoverin), a suitable animal species has to be selected for this purpose. These may include rabbits, which are highly susceptible to posterior uveitis and are similar to humans in terms of the biomechanical and biochemical properties of the visual system [24,25,26,27]. Effective targeting of immune responses in patients should be based on an understanding of the biochemical alterations associated with uveitis, particularly the patterns of specific immune effectors. The screening for such effectors can be accomplished in EAU models by analyzing aqueous humor (AH), which maintains physiological, nutritional and metabolic homeostasis of the ocular tissues. Indeed, AH composition is largely affected by ocular inflammation, and its testing is widely used to assess inflammatory mediators, such as cytokines and chemokines, in autoimmune uveitis in humans and animal models of posterior uveitis [28,29,30,31,32,33,34]. Moreover, AH can be employed for monitoring cellular responses in uveitis, as, in EAU, it becomes infiltrated by activated T lymphocytes [34]. Our recent omics studies have shown that mediators of ocular inflammation include signaling lipids (phospholipid derivatives, polyunsaturated fatty acids (PUFAs) and oxylipins) that are well known as convenient targets for pharmacotherapy [26,35,36,37,38]. Large-scale screening of such molecules in non-infectious autoimmune uveitis by targeted metabolomics has never been performed.

Here, we demonstrated for the first time that recoverin is an uveoretinal antigen in albino rabbits, as immunization with this protein causes typical autoimmune chorioretinitis. The pathological process induced by recoverin has been extensively studied using clinical and histological approaches, as well as monitoring of retinal electrophysiological activity. In addition, the mechanisms in EAU have been characterized by analyzing oxidative stress markers, key cytokines and zinc content in AH and, for the first time, by resolving its metabolome and signaling lipidome. The results of these studies propose new lines of therapy for autoimmune uveitis, one of which was validated using the developed model.

## 2. Results

### 2.1. Recoverin-Induced EAU: Clinical Characteristics and Antibody Response

**Figure 1:**
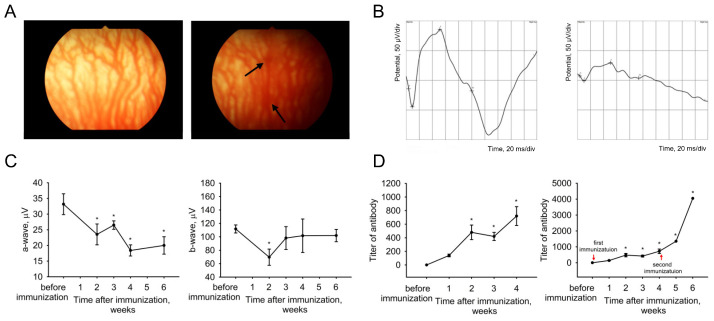
Clinical characterization of recoverin-induced EAU in rabbits. (**A**) Representative photographs of the ocular fundus before (left) and 4 weeks after (right) immunization with recombinant bovine recoverin (7.5 mg/mL). Arrows indicate subretinal hemorrhages. (**B**) Scotopic ERG recordings performed before the immunization (left) and at week 4 after the procedure (right). (**C**) Amplitudes of the a-wave (left) and b-wave (right) of the electroretinogram at different time periods after recoverin administration. (**D**) Accumulation of antibodies against recoverin in sera according to ELISA. * *p* < 0.05 compared to the values obtained before immunization.

To induce autoimmune uveitis, albino white rabbits were immunized with a homogeneous preparation of recombinant bovine recoverin mixed with complete Freund’s adjuvant. The pathological process was monitored by fundoscopy and electroretinography, which were performed before immunization and once a week for six subsequent weeks. In addition, the dynamics of antibody production against recoverin was analyzed in animals` sera by ELISA (Figure 1). The development of a characteristic clinical picture of posterior uveitis was observed within four weeks after immunization. During this period, fundoscopy showed no signs of ocular media opacity, retinal detachment or optic nerve edema, but by the fourth week, signs of bifurcation of the choroidal vessels and apparently subretinal hemorrhages became apparent (Figure 1A). In parallel, a gradual but stable decrease in the amplitude of the a-wave of the electroretinogram was observed, indicating impaired functioning of photoreceptor cells, while the amplitude of the b-wave (reflecting the activity of bipolar cells) significantly declined only in the second week after immunization (Figure 1B,C). No further changes in retinal activity were observed at weeks 5 and 6. ELISA of the rabbit sera demonstrated that the above processes were accompanied by a gradual accumulation of antibodies, the titer of which reached 1:728 by the end of the fourth week (Figure 1D, left panel). To optimize the conditions for the EAU induction, some animals were reinjected with recoverin four weeks after the initial immunization. Reimmunization predictably stimulated antibody production but without additional clinical and electroretinographic alterations (Figure 1D, right panel). Overall, a single immunization of albino rabbits with recoverin induces posterior autoimmune uveitis manifested within four weeks as chorioretinitis with decreased electrophysiological activity of the retina and humoral immune response.

### 2.2. Recoverin-Induced EAU: Morphological Characteristics

**Figure 2:**
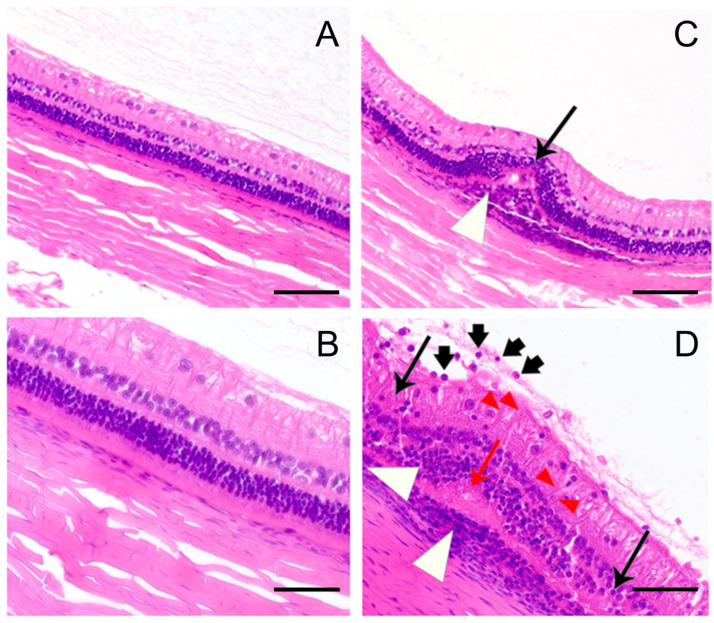
Histological characterization of recoverin-induced EAU in rabbits. Morphology of the posterior sector of the eye before (**A**,**B**) and 4 weeks after (**C**,**D**) immunization with recombinant bovine recoverin (7.5 mg/mL in PBS) mixed with Freund’s complete adjuvant (1:1). Inflammatory mononuclear cells infiltrating the vitreous body and retina (**D**, short black arrows), Dalen–Fuchs nodules (**C**,**D**, white arrows), the swelling of photoreceptors up to their fusion into homogeneous eosinophilic droplets (**D**, red arrows), activation of the Müller glia (**D**, red arrowheads) and areas of retinal atrophy (**C**,**D**, long black arrows) are indicated. Retinal thickness in images (**C**,**D**) is altered relative to images (**A**,**B**) due to inflammatory and atrophic changes. Retinal fissures and detachment are of artificial origin. Staining by hematoxylin and eosin. Magnification: 200× (top row) or 400× (bottom row). Scale bar 100 µm on (**A**) (top row) and 50 µm on (**B**) (bottom row).

To characterize the pathomorphological changes associated with recoverin-induced uveitis, certain animals were euthanized four weeks after recoverin administration, and the posterior sector of the enucleated eyes was subjected to histological examination (Figure 2). The most common sign observed in the experimental preparations was focal infiltration of the retina with inflammatory mononuclear cells (Figure 2D). In some areas, the infiltration was revealed in the adjacent vitreous body and choroid, in which Dalen–Fuchs nodules were detected (Figure 2C,D). These nodules are both a histologic and clinical manifestation of sympathetic ophthalmia and most commonly represent granuloma-like clusters of epithelioid cells, lymphocytes and histiocytes covered by an intact layer of retinal pigment epithelium [39,40]. Thus, the retina underwent infiltration by inflammatory cells from both the choroidal side and the vitreous side. In the areas of the most pronounced lesions, swelling of the photoreceptors up to their fusion into homogeneous eosinophilic droplets was observed (Figure 2D). Given that recoverin is a photoreceptor protein, this phenomenon can be regarded as one of the stages of autoimmune damage of these cells as a result of immunization. The involvement of the choroid and retina was heterogeneous: in the most severe areas, the retina underwent subtotal or total atrophy (Figure 2C,D). Notably, the retinal thickness did not correlate directly with the severity of the lesion: in areas of mild inflammation, it remained unchanged, and in moderate inflammation, it thickened due to edema and infiltration, while, in severe inflammation, it significantly thinned due to atrophy. It should be added that the inflamed retina was characterized by activation of the Müller glia, morphologically expressed in thickening of its eosinophilic outgrowths (Figure 2D). In summary, the revealed phenomena of retinal and vitreous body infiltration, photoreceptor atrophy and Dalen–Fuchs nodule formation corresponded to the typical picture of chronic autoimmune chorioretinitis.

### 2.3. Recoverin-Induced EAU: Biochemical Alterations

#### 2.3.1. Inflammation and Oxidative Stress Markers

Since the developed EAU clinically and histologically reproduced the human disease well (see the Section 3), we further investigated its biochemical characteristics to better understand the underlying mechanisms. As mentioned above, ocular inflammation is reflected on the composition of AH, which can be used to analyze the molecular mediators of autoimmune uveitis. Therefore, we collected AH from animals before (control samples) and 2–4 weeks after immunization with recoverin (experimental samples) and performed biochemical analysis, comparing the concentrations of various AH components in these control and experimental samples. 

**Figure 3:**
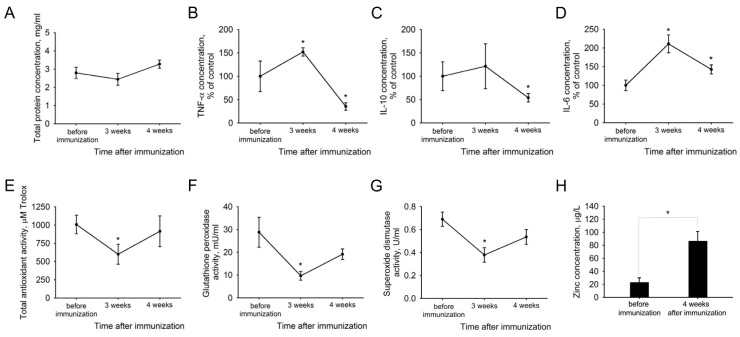
Biochemical changes in AH during the development of recoverin-induced EAU in rabbits. AH was collected before (control samples) and 3 and 4 weeks after immunization of the animals (experimental samples) using recombinant bovine recoverin (7.5 mg/mL in PBS mixed with Freund’s complete adjuvant 1:1) and analyzed for the total protein concentration (**A**), cytokine content (**B**–**D**), total antioxidant activity (**E**), antioxidant enzymes activities (**F**,**G**) and zinc level (**H**). * *p* < 0.05 compared to the values obtained for the control samples (before immunization).

Firstly, the samples were analyzed for macro signs of inflammation, as well as key proinflammatory cytokines TNF-α and IL-6 and immunosuppressive mediator IL-10. As inflammation is often accompanied by oxidative stress, AH was also analyzed for its markers, such as total antioxidant capacity and the activity of key antioxidant defense enzymes superoxide dismutase (SOD) and glutathione peroxidase (GPx). Inflammatory changes in AH were generally moderate and do not include severe symptoms such as infiltration by inflammatory cells or an increase in total protein (Figure 3A). The most significant changes were observed in the third week after immunization and included increases in TNF and IL-6 (Figure 3B,D), and decreases in all components of antioxidant defense (Figure 3E–G). Decreased levels of the anti-inflammatory mediator IL-10 were observed in the later phase of EAU (week 4, Figure 3C). Oxidative stress was accompanied by a zinc accumulation in AH, which, according to atomic absorption spectroscopy (AAS) data, reached a four-fold elevation at week 4 (Figure 3H). Thus, oxidative stress and TNF-α/IL-6-mediated proinflammatory signals are characteristic features of recoverin-induced EAU that precede and accompany the development of structural and functional changes in the retina.

#### 2.3.2. Signaling Lipidome

**Figure 4:**
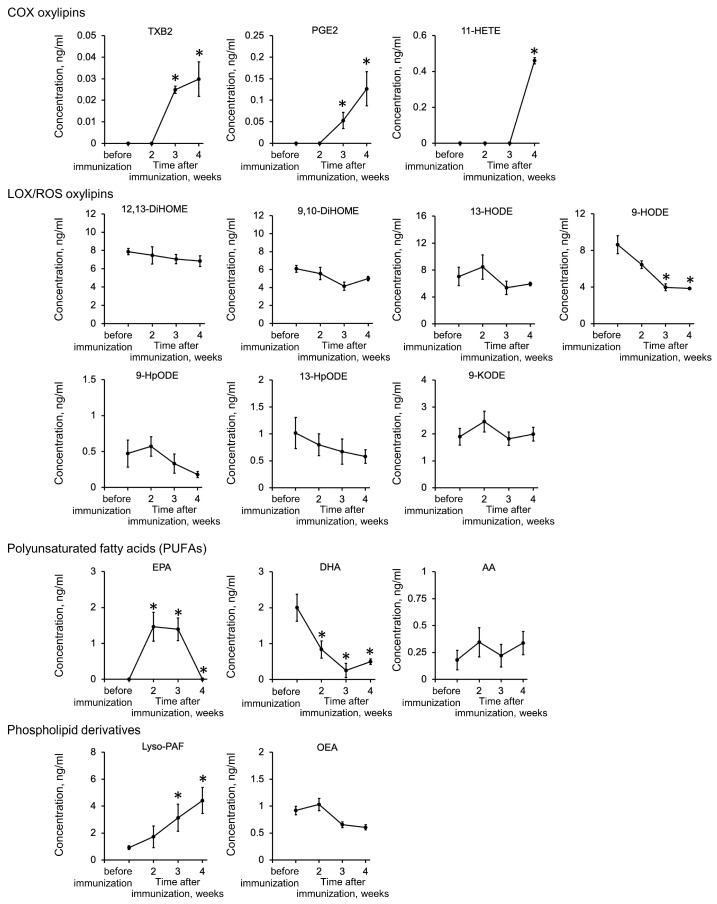
Changes in the lipid composition of AH during the development of recoverin-induced EAU. AH was collected before (control samples) and 3 and 4 weeks after immunization of the animals (experimental samples) using recombinant bovine recoverin (7.5 mg/mL in PBS mixed with Freund’s complete adjuvant 1:1) and analyzed for the signal lipid content. The identified lipid mediators were grouped according to their chemical families (PUFAs, oxylipins and phospholipid derivatives). Oxylipins were further classified according to their biosynthesis pathways involving COX, LOX or CYP. * *p* < 0.05 compared to the values obtained for the control samples (before immunization).

It was reported that inflammation in EAU can be regulated involving PUFA-derived lipid mediators such as PGE2 (prostaglandin E2) and LTC4 (leukotriene C4), the altered contents of which were found in AH [41,42,43]. To recognize the signaling lipid-dependent mechanisms in recoverin-induced EAU and to confirm the feasibility of this model to test anti-inflammatory and antioxidant therapies, we performed a full-scale screening and comparison of such molecules in AH collected from a separate group of animals before (control samples) and 2, 3 and 4 weeks after immunization (experimental samples) using the UPLC-MS/MS-based targeted lipidomics method developed in our previous works. A total of three PUFAs (AA, DHA and EPA); 10 oxylipins, including derivatives of AA (TXB2, PGE2 and 11-HETE) and LA (9-HpODE/9-HODE/9-KODE, 13-HpODE/13-HODE, 9,10-DiHOME and 12,13-DiHOME) and one phospholipid derivative (lyso-PAF) were identified (Figure 4). Quantitative analysis revealed altered patterns of inflammatory mediators at 3–4 weeks after immunization. Primarily, it is the increase in the level of key proinflammatory molecules, including cyclooxygenase (COX)-dependent AA derivatives (TXB2, PGE2 and 11-HETE) and Lyso-PAF, which is the PLA2-dependent precursor of PAF (platelet-activating factor), a potent phospholipid activator of inflammation. Secondly, it is the decrease in levels of the key anti-inflammatory molecule, the omega-3 PUFA DHA. Another anti-inflammatory PUFA, EPA, showed an increase at weeks 2–3, which, however, was completely reversed by week 4. Thirdly is the downregulation of LOX-dependent oxylipin 9-HODE, which belongs to pleiotropic bioactive compounds, oxidized metabolites of LA (OXLAMs). It is noteworthy that 11-HETE is not only the byproduct of prostaglandin biosynthesis via the COX pathway but can also be synthesized as a result of non-enzymatic oxidation and is generally recognized as a marker of lipid peroxidation [38,39]. Taken together, these data suggest a role for oxidative stress, as well as AA/COX and PAF-dependent pathways, in the ocular inflammation characteristic of recoverin-induced EAU.

#### 2.3.3. Metabolome

**Figure 5:**
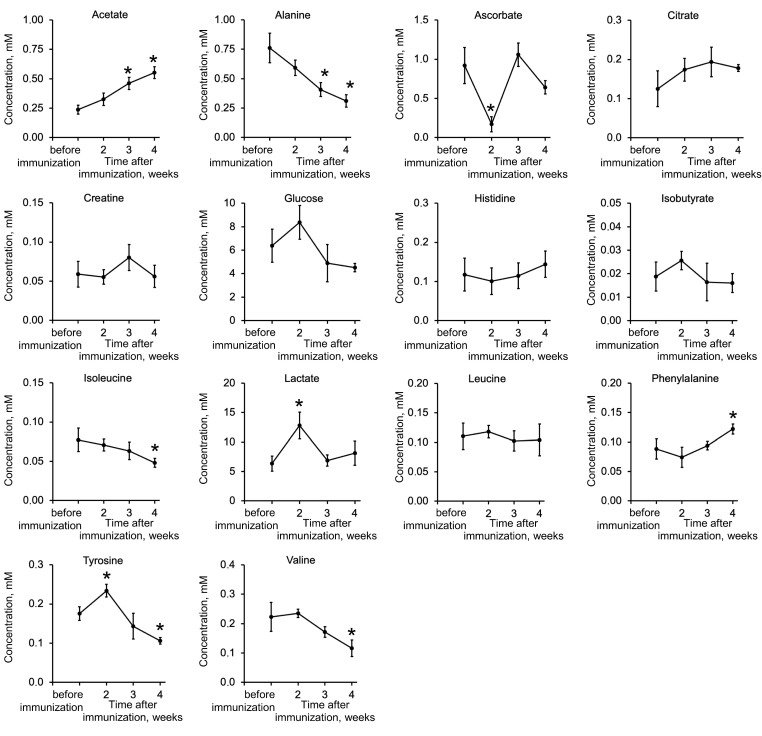
Changes in AH metabolome during the development of recoverin-induced EAU. AH was collected before (control samples) and 3 and 4 weeks after immunization of the animals (experimental samples) using recombinant bovine recoverin (7.5 mg/mL in PBS mixed with Freund’s complete adjuvant 1:1) and analyzed for core metabolites. The identified compounds are presented in alphabetical order. * *p* < 0.05 compared to the values obtained for the control samples (before immunization).

Local biochemical changes characteristic of autoimmune uveitis can be recognized by analysis of the AH metabolome. Indeed, alterations in metabolic pathways reflected in the AH composition have been found in Vogt–Koyanagi–Harada disease, Behçet’s disease and Fuchs syndrome [44,45]. Given these observations, we next analyzed the corresponding changes in EAU induced by recoverin. To address this issue, we determined and compared the core metabolomic profiles of AH collected before (control samples) and 2, 3 and 4 weeks after immunization (experimental samples) using NMR spectroscopy. A total of 14 compounds were identified, including seven amino acids (alanine, histidine, isoleucine, leucine, phenylalanine, tyrosine and valine) and four organic acids (acetate, citrate, isobutyrate and lactate), as well as glucose, ascorbic acid and creatine (Figure 5). Only eight of them showed significant changes during the development of EAU, namely alanine, isoleucine, phenylalanine, tyrosine, valine, acetate, lactate and ascorbate. There was a general trend of decreasing alanine, valine and ascorbic acid while increasing phenylalanine, tyrosine, acetate and lactate. The characteristic time point was the second week after immunization, when there was a significant decrease in ascorbic acid accompanied by an increase in lactate and tyrosine as compared to their concentrations before immunization. Overall, we concluded that EAU significantly affects amino acid metabolism and homeostasis of ascorbic acid, which is a key antioxidant of AH.

#### 2.3.4. Recoverin

**Figure 6:**
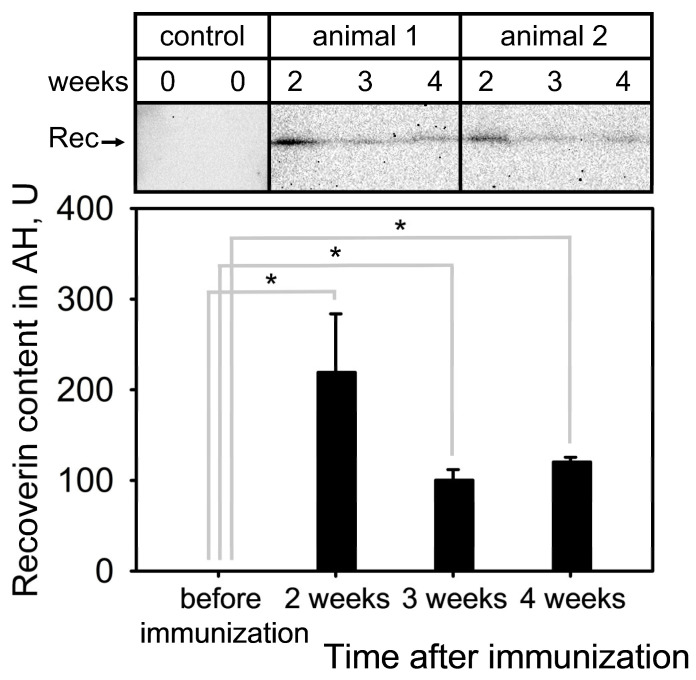
Leakage of recoverin into AH during EAU development. AH was collected before (control samples) and 3 and 4 weeks after immunization of the animals (experimental samples) using recombinant bovine recoverin (7.5 mg/mL in PBS mixed with Freund’s complete adjuvant 1:1) and analyzed for the presence of recoverin (Rec) by Western blotting (top). The amounts of recoverin in AH were calculated by densitometric analysis of the protein bands (bottom). * *p* < 0.05 compared to the values obtained for the control samples (before immunization).

A key hallmark of autoimmune uveitis is disruption of the blood–ocular barrier, which can lead to the penetration of recoverin into AH from both blood and/or damaged photoreceptor cells via the vitreous-to-aqueous gradient [46,47,48]. Therefore, to follow the state of the barrier during EAU induced by recoverin, we determined its content in AH samples collected before, as well as 2, 3 and 4 weeks after, immunization by Western blotting. Only trace amounts of recoverin were detected in the control samples of AH (before immunization, Figure 6). However, the protein level increased sharply in the second week, then decreased two-fold but remained noticeable until the fourth week after immunization. These observations suggest that the blood–retinal and/or blood–aqueous barriers were disrupted early in the EAU and remained permeable until the fourth week, allowing photoreceptor antigens to leak into the extracellular space and AH. This can exacerbate the autoimmune process and also may lead to the formation of aberrant complexes of these antigens with secreted extracellular proteins, possibly affecting their function.

### 2.4. Recoverin-Induced EAU: Suppressing Oxidative Stress and Inflammation by Mitochondria-Targeted Antioxidant

**Figure 7:**
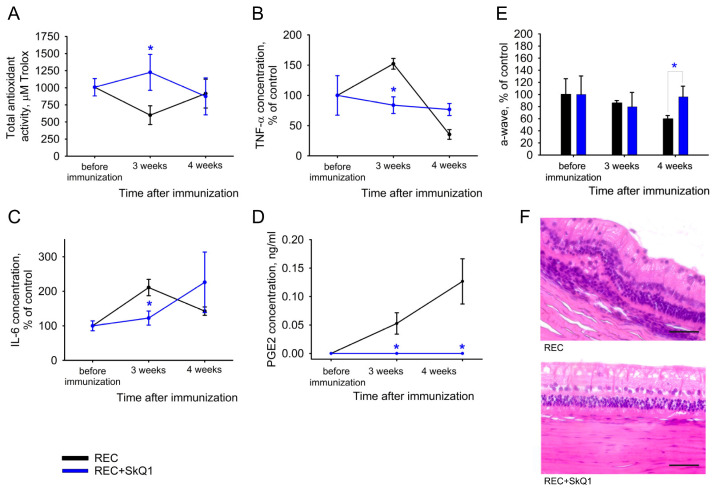
Parameters of recoverin-induced EAU under mitochondria-targeted antioxidant therapy. Treatment with 7.5 μM SkQ1 was performed 3 times daily for 3 days before immunization and 3 times daily for 3 or 4 weeks after immunization with recoverin. AH was collected in the groups with or without treatment before and 3 and 4 weeks after immunization. The samples were subjected to biochemical examination, as described in Figure 3 and Figure 4. Results of the analysis of antioxidant activity (**A**), as well as TNF-α (**B**), IL-6 (**C**) and PGE2 (**D**) concentrations, are presented. (**E**,**F**) Scotopic ERG recordings (**E**) and morphology of the posterior sector of the eye (**F**; hematoxylin and eosin staining, magnification 400×, scale bar 50 μm) at week 4 after immunization with or without treatment. * *p* < 0.05 compared to the corresponding values obtained for samples from the group without treatment.

Our previous data showed that the development of light-induced retinal degeneration (LIRD) in rabbits (model of AMD [16,46]) can be effectively prevented by treatment with mitochondria-targeted antioxidant SkQ1, which strongly protects the retinal structure and electrophysiological activity by exhibiting both antioxidant and anti-inflammatory effects, including targeting AA/COX-dependent pathways [36,49]. Given these data and our current results, we evaluated the therapeutic efficacy of this drug against recoverin-induced EAU to validate the applicability of this model for testing anti-inflammatory and antioxidant therapies. Conjunctival instillations of SkQ1-containing eye drops were performed for 3 days before immunization with recoverin (premedication) and for 3 or 4 weeks after the immunization (treatment). Oxidative stress and inflammation were monitored by biochemical and lipidomic analysis of AH, as well as histologic examination of the posterior segment of the eyes. Administration of SkQ1 prevented the decrease in antioxidant activity of AH without significant effect on antioxidant defense enzymes and completely suppressed the increase in TNF-α and IL-6 (Figure 7A–C). Moreover, the treatment reduced PGE2 levels at the third week after immunization (Figure 7D), although no effect on other oxylipins or lyso-PAF was detected. Unexpectedly, we found almost no histologic evidence of uveitis in the chorioid, retina or vitreous, nor electrophysiologic changes in retinal activity, in any of the SkQ1-treated animals (Figure 7E,F). Despite the fact that, due to the multifocal nature of the pathological changes in autoimmune uveitis, the presence of individual unrecognized lesions could not be completely excluded; in general, the administration of mitochondrial-targeted antioxidant therapy produced a pronounced anti-inflammatory and retinoprotective effect, demonstrated in a case of autoimmune uveitis for the first time.

## 3. Discussion

Previous studies reported uveitogenic activity of recoverin in Lewis rats and identified peptides eliciting a response in this species [17,18,50]. Here, we demonstrated for the first time that recoverin is an uveoretinal antigen in albino rabbits, a species that is more similar to humans in the biochemical and biomechanical properties of the visual system and much more suitable for ophthalmic drug trialing [25]. Immunization of rabbits with recoverin induces a more rapid development of EAU compared to other retinal antigens. Thus, the main symptoms up to retinal damage manifest within 4 weeks, while, for example, after SAG administration, immune cell infiltration is observed after 2–3 weeks, and photoreceptor degeneration develops only from week 4 to 7 [51]. Recoverin-induced EAU shares many features with various forms of human autoimmune uveitis, such as birdshot chorioretinopathy, Vogt–Koyanagi–Harada disease or sympathetic ophthalmia [4,52,53]. Shared histopathologic signs include lymphocytic infiltration, Dalen–Fuchs nodules between the RPE and Bruch’s membrane and foci of subtotal or total retinal atrophy, especially of the outer retinal layers [4,52,53]. The loss of photoreceptors in human disease is manifested by a decrease in retinal electrophysiologic activity [4,52,53,54], and our model also showed a decrease in scotopic ERG a-wave amplitude. Occasional findings in human posterior uveitis are subretinal hemorrhages [55,56], which were evident in recoverin-induced EAU. Given that antibodies to recoverin are often detected in the blood of patients with autoimmune uveitis [23], EAU induced by this protein in rabbits seems be one of the most accurate models of the human disease.

Similarities with human autoimmune uveitis were also noted in the biochemical analysis of rabbit AH, which furthermore allowed identifying the key signaling pathways that control the inflammatory process and can be targeted by pharmacotherapy. Thus, idiopathic non-infectious uveitis in humans is characterized by increased TNF-α, IL-6, IL-2 and INF-γ and decreased IL-10 in AH [32], and we observed a similar trend for TNF-α, IL-6 and IL-10 in recoverin-induced EAU. Moreover, autoimmune uveitis in patients may be associated with polymorphisms of *TNF* and *IL10* genes [32,57]. TNF-α and IL-6 are indeed recognized as important mediators of ocular inflammation in the early stages of autoimmune uveitis. IL-6 activity is at least partially realized through the induction of Th17 differentiation, while TNF-α contributes to BRB disruption [58,59]. Consistently, knockout of IL6 or TNFR1 genes in mice results in resistance to EAU, and the administration of antibodies against the TNF-α or IL-6 receptor alleviates the course of the disease [58,59,60,61]. It has been previously shown that, in EAU, local IL-6 production reduces the immunosuppressive properties of the ocular microenvironment, leading to the spread of inflammation, which, at later stages, is resolved with the participation of regulatory T and B cells secreting IL-10 [3,33]. The cytokine profile of recoverin-induced EAU identified in our work generally confirms these mechanisms.

Idiopathic autoimmune uveitis is characterized by disruption of the blood–ocular barrier and progressive photoreceptor destruction [62], which can lead to leakage of photoreceptor proteins into the extracellular space and AH. Extracellular leakage of recoverin was observed in the rabbit model of LIRD characterized by massive disintegration of the photoreceptor cells [16,46]. The released recoverin can reach AH, which accumulates factors secreted by the retina due to the vitreous-to-aqueous gradient [47,48]. Indeed, recoverin was recognized as one of the unique proteins present in vitreous fluid of patients with noninfectious uveitis [63]. Consistently, in the present study, we observed an increase in the amount of recoverin in the AH of rabbits with EAU. In this case, recoverin can enter the AH from both the retina and the blood due to the disruption of the blood–aqueous barrier. Aberrant extracellular localization of recoverin may exacerbate the immune response and also affect local signaling pathways in the retina. For instance, neuronal calcium sensors can form high-affinity complexes with IL-11 [64], an anti-inflammatory cytoprotective cytokine secreted by RPE cells in response to TNF-α [65], elevated levels of which are a hallmark of our EAU model. Released recoverin could similarly modulate cytokine signaling or the function of other secreted proteins, which may contribute to the pathogenesis of autoimmune uveitis. In addition, due to accumulation in the AH, recoverin can be regarded as a biomarker of EAU and possibly other ocular diseases associated with photoreceptor damage.

Increasing evidence suggests that intraocular inflammation in various ophthalmic diseases is regulated by signaling lipids such as PUFAs and their derivatives oxylipins, which can be monitored in AH [26,35,36,38]. However, studies of such molecules in human autoimmune uveitis have never been conducted. Thus, using our EAU model, we have provided a first description of the signaling lipidome of AH in this disease. It was demonstrated that the inflammatory process is associated with an increase in primarily COX-dependent derivatives of AA, namely PGE2, TXB2 and 11-HETE. These observations are in line with previous data obtained for SAG-induced EAU, which is also characterized by an elevated PGE2 content [40,41,42]. Consistently, EAU can be partially suppressed by topical nonsteroidal anti-inflammatory drugs inhibiting COX-2 and preventing BRB breakdown [66]. Another important signaling molecule significantly upregulated in recoverin-induced EAU is lyso-PAF, which is a PLA2-dependent precursor of PAF (platelet-activating factor), a potent phospholipid activator of inflammation [67]. The specific role of PAF in EAU is supported by the fact that, when administered intravitreally to rats or rabbits, it induces retinitis without causing anterior uveitis [68]. Finally, two molecules exhibited downregulation in recoverin-induced EAU, namely the omega-3 PUFA DHA and LOX-dependent oxylipin 9-HODE, which belong to pleiotropic bioactive compounds, oxidized metabolites of LA (OXLAMs). Under normal conditions, DHA is highly enriched in the retina and exhibits neuroprotective and anti-inflammatory activities [69]. Diet supplementation of DHA suppresses IRBP-induced EAU in mice via inhibiting the activity of Th1 and Th17 cells [70]. Correspondingly, the drop in DHA content found in our model may be a key component of its pathogenesis. It should be added that the pattern of oxylipins in recoverin-induced EAU is similar to that we found in AH of rabbits with the model of LIRD, revealing common signaling mechanisms of retinal inflammation [36].

It is important to note that 11-HETE, which is markedly increased in recoverin-induce EAU, is often synthesized by non-enzymatic oxidation, thus being a marker of lipid peroxidation, one of the manifestations of oxidative stress [38,39]. In fact, in a biochemical analysis of AH, we observed a number of signs of oxidative stress, the main one being a decrease in total antioxidant activity caused by the consumption of low molecular weight antioxidants. This trend was well confirmed by our metabolomic studies, which registered an increase in lactate and decrease in ascorbic acid, which are known as markers of oxidative stress and the major antioxidant in AH, respectively [71,72], in the early stage of EAU. The identified amino acid signature of AH, namely upregulation of tyrosine and phenylalanine in the early stage and downregulation of branched-chain amino acids in the advanced stage, may also indicate the activation of redox-regulated pathways [73,74]. Similar changes, such as decreased levels of lactic acid and valine and increased levels of tyrosine and alanine, were found in AH patients with Behçet’s disease and Vogt–Koyanagi–Harada syndrome [44]. Oxidative stress in ocular tissues may also be accompanied by zinc accumulation in AH released from Zn^2+^-buffering proteins metallothioneins in response to their oxidation [75]. Indeed, increased AH zinc has been found in common retinal diseases associated with oxidative stress, such as AMD and glaucoma [76,77]. Consistently, we found a four-fold increase in AH zinc in late-stage EAU, which is the first demonstration of such an effect in uveitis to the best of our knowledge. The disrupted zinc homeostasis makes zinc a pathological factor causing neurodegenerative changes in the retina [78], which, based on our data, may also occur in posterior uveitis. Oxidative stress is indeed one of the most important driving forces of autoimmune uveitis. Oxidative damage to the retina in EAU is associated with the actions of both innate and adaptive immunity, with the former involving activation of toll-like receptors that increase inflammatory cytokines, leading to oxidative stress, and the latter involving increased inflammatory cytokines and mitochondrial oxidative stress [79]. Mitochondrial DNA damage, downregulation of mitochondrial proteins and suppression of mitochondrial function become evident in the early stages of EAU. A similar mechanism in sympathetic ophthalmia leads to oxidative damage of the photoreceptors [79].

As was mentioned above, the developed model of recoverin-induced EAU can be used to search for treatment approaches for noninfectious posterior uveitis. Indeed, recent advances in ocular pharmacology have opened up new applications for rabbit models of EAU that can be used to study new drugs with different routes of administration, including topical drugs, Sub-Tenon injections, suprachoroidal injections, intravitreal injections and surgical implants [25,80]. Our findings on the role of oxidative stress in EAU suggest that optimization of the treatment regimen can be achieved by incorporating antioxidant therapy. Given the mitochondrial origin of oxidative stress in noninfectious uveitis, targeting these organelles with antioxidants appears to be the most effective way to potentiate therapy. This assumption is supported by the results of the present work, which showed that premedication/treatment with the mitochondria-targeted antioxidant SkQ1 prevented retinal damage, at least in the analyzed sites. Previously, SkQ1 demonstrated high efficacy in preventing oxidative damage of the retina in the LIRD model [36,49,81], indicating the high bioavailability and overall retinoprotective potential of this drug. In addition to mitochondria-targeted antioxidant therapy, our results suggest a number of potential therapeutic options for autoimmune uveitis, including blockade of TNF-α/IL-6 and PAF signaling, inhibition of prostaglandin synthesis or DHA and ascorbic acid supplementation. The new EAU model presented here can be used to test individual or combined versions of these or other treatments for posterior uveitis.

## 4. Materials and Methods

### 4.1. Materials

Recombinant myristoylated bovine recoverin (UniProt #P21457) was obtained as described in our recent work [82]. The molecular weight and homogeneity of the protein were confirmed by SDS-PAGE, C18 reverse phase HPLC and mass spectrometry, and its purity exceeded 95%. Ophthalmic drops containing SkQ1 (10-(60-plastoquinonyl)-decyltriphenylphosphonium) were provided by the Institute of Mitoengineering of Moscow State University (Moscow, Russia). Tiletamine and zolazepam were from Virbac (Carros, France). Xylazine hydrochloride was from Nita-Farm (Saratov Oblast, Russia). Phosphate-buffered saline (PBS) was from Thermo Fisher Scientific/Gibco (Waltham, MA, USA). TNF-α, IL-10 and IL-6 assay kits were from Cloud-Clone Corp. (Katy, TX, USA). Hemoglobin, luminol, hydrogen peroxide and Trolox were from Sigma-Aldrich (Saint Louis, MO, USA). Zn(NO_3_)_2_ and Mg(NO_3_)_2_ were from Fluka (USA); EDTA was from Amresco (St. Louis, MO, USA). Reagents for the histological examination were from Biovitrum (Moscow, Russia). The bicinchoninic acid assay kit was from Sigma-Aldrich (USA). Oasis^®^ PRIME HLB cartridge (60 mg, 3cc, cat. no. 186008056) was obtained from Waters (Eschborn, Germany). Oxylipins standards for mass-spectrometry tetranor-PGEM-d6 (cat. no. 314840), 6-keto PGF1α-d4 (cat. no. 315210), TXB2-d4 (cat. no. 319030), PGF2α-d4 (cat. no. 316010), PGE2-d4 (cat. no. 314010), PGD2-d4 (cat. no. 312010), Leukotriene (LT) C4-d5 (cat. no. 10006198), LTB4-d4 (cat. no. 320110), 5(S)-HETE-d8 (cat. no. 334230), 12(S)-HETE-d8 (cat. no. 334570), 15(S)-HETE-d8 (cat. no. 334720), PAF C16-d4 (cat. no. 10010229), Oleoyl Ethanolamide-d4 (cat. no. 9000552) and PGA2-d4 (cat. no. 310210) were from Cayman Chemical (Ann Arbor, MI, USA). Optima™ LC/MS Grade methanol was from Thermo Fisher Scientific/Fisher Chemical (Waltham, MA, USA). DSS-d6 was from Cambridge Isotope Laboratories (Andover, MA, USA). Other reagents were from Sigma–Aldrich, Amresco (St. Louis, MO, USA) or Serva (Heidelberg, Germany). All buffers were prepared using ultrapure deionized water.

### 4.2. Animals and Ethics Statement

Male albino rabbits aged 6 months weighing from 2.3 to 3 kg (Manihino, Moscow, Russia) were used. The animals were kept in individual cages under a 12-h light and dark cycle at 22–25 °C and 55–60% humidity with free access to feed and water. The health status of the animals was monitored daily, and no adverse events were observed. AH collection was performed under general anesthesia by intramuscular injection of a 1:2 mixture of 50 mg/mL tiletamine/zolazepam and 20 mg/mL xylazine hydrochloride. Prior to AH sampling, the animals were additionally anesthetized locally with Alcaine. For histological analysis of the eyes, the animals were humanely euthanized with an anesthetic overdose. Enucleation of the eyeballs was performed postmortem. All procedures were performed according to the 8th edition “Guide for the Care and Use of Laboratory Animals” of the National Research Council and “Statement for the Use of Animals in Ophthalmic and Visual Research” of The Association for Research in Vision and Ophthalmology (ARVO). The protocol was approved by the Belozersky Institute of Physico-chemical Biology Animal Care and Use Committee (Protocol number 1/2016).

### 4.3. Induction, Clinical/Electrophysiological Evaluation and Treatment of EAU

For induction of autoimmune uveitis, rabbits were immunized with a solution containing 7.5 mg/mL of recombinant bovine recoverin in PBS mixed with Freund’s complete adjuvant (1:1). The resulting emulsion was administrated subcutaneously, 6 injections along the spine. The development of posterior uveitis was assessed by clinical examination, and ERG was performed before immunization and once a week for 6 weeks after immunization with recoverin. Clinical analysis was conducted using a slit lamp (Heine, Herrsching am Ammersee, Germany) and fundus camera (Kowa Genesis-D, Tokyo, Japan). Any possible changes in the cornea, lens, vitreous body, retina and optic nerve were evaluated. ERG was recorded in the standard scotopic mode (animals were dark-adapted for 1 h before analysis) under anesthesia and mydriasis (1% tropicamide) using the RETIport ERG electrophysiologic system (An-vision, Hennigsdorf, Germany). Recordings were made in response to white LED (7000 K) flashes of 3 cd∙s/m^2^ intensity and 0.3 Hz frequency. To monitor antibody production, blood samples (1 mL) were collected before immunization and once a week thereafter (1–6 weeks) from the marginal vein of the auricle through an intravenous catheter (24 G) into a sterile tube with clotting activator. After centrifugation (2500 rpm, 30 min), the supernatant (serum) was analyzed for anti-recoverin antibodies by standard ELISA using recombinant bovine recoverin as the antigen. In the groups to trial mitochondria-targeted therapy, rabbits received conjunctival instillations of 7.5 μM SkQ1 for 3 days before immunization (premedication) and for 3 or 4 weeks after immunization (treatment). AH for biochemical analysis was collected as described above before immunization (control samples) and at 2–4 weeks after immunization (experimental samples).

### 4.4. Histological Evaluation of EAU

Histological analysis of the posterior sector of the eye was performed in selected animals 4 weeks after immunization. Animals were euthanized, and eyeballs were enucleated and placed for 3 h in freshly prepared Carnoy’s fixative (60% ethanol, 30% chloroform and 10% glacial acetic acid). After fixation, eyeball specimens were washed with 96% ethyl alcohol, sectioned into 2 pieces along the nasotemporal axis, dehydrated in increasing concentrations of ethanol and xylene in an automated tissue processor and embedded in paraffin. Six to eight representative 3–4 μm thick paraffin sections were obtained from each eye. Sections were mounted on slides and stained with hematoxylin and eosin. Histological preparations were analyzed using a Zeiss Axio Observer A1 light microscope (Carl Zeiss, Oberkochen, Germany). Representative microphotographs were obtained using an AxioCam 305 high-resolution digital microscopic camera. Microphotographs were processed using Zeiss Zen 2 lite blue edition software (Carl Zeiss) and Adobe Photoshop CS6 (Adobe, San Jose, CA, USA).

### 4.5. Biochemical Characterization of AH in EAU

The total antioxidant capacity (TAC) of AH was analyzed according to the method developed in our previous studies [36,49]. The reaction mixture (500 μL) contained AH (1:200 in PBS) or 1–8 μM Trolox, 0.01 mM luminol and 0.5 μM hemoglobin in PBS. The mixture was incubated for 3 min at 37 °C, and the reaction was initiated by adding H_2_O_2_ to a final concentration of 6 μM. Chemiluminescence was recorded at a wavelength of 425 nm for 600 s using a Glomax-Multi Detection System luminometer (Promega, Madison, WI, USA). TAC was expressed in Trolox equivalents. GPx and SOD activities were determined with #RS504 and #SD125 colorimetric kits (Randox, Crumlin, County Antrim, UK). TNF-α, IL-10 and IL-6 were assayed by ELISA kits (Cloud-Clone Corp., Katy, TX, USA). All colorimetric reactions were monitored using a CLARIOstar Plus reader (BMG Labtech, Ortenberg, Germany). The content of recoverin was evaluated by Western blotting of AH samples using rabbit polyclonal anti-recoverin antibodies (#DF3160, Affinity Biosciences, China; 1:1000 in PBST) and rat polyclonal anti-recoverin antibodies prepared as described in our previous study [20] (1:10,000 in PBST). The protein bands were detected using horseradish peroxidase-conjugated anti-rabbit IgG antibody (Jackson Immunoresearch, West Grove, PA, USA) and anti-rat IgG antibody (#sc-2065, Santa Cruz Biotechnology, PA, USA; 1:5000 in PBST) and visualized using Clarity Western Enhanced Chemiluminescence (ECL) Substrate and the ChemiDoc™ XRS+ gel documentation system (Bio-Rad, Hercules, CA, USA). The amounts of the proteins in AH were calculated by densitometric analysis of the bands using GelAnalyzer.2010a software (http://www.gelanalyzer.com/). Zinc content in AH was measured using an atomic absorption spectrometer with electrothermal atomization and Zeeman background correction (iCE 3000, Thermo Fisher Scientific, USA) at 213.8 nm. Calibration was performed in the range of 0.5–10 μg/L Zn^2+^ using standard solutions of Zn(NO_3_)_2_. The measurements were performed in the presence of 0.5 mM Mg(NO_3_)_2_ and 0.5 mM EDTA to reduce the interference effect of chloride ions. Each AH sample was examined in a series of 3–6 parallel dilutions and the absorption signal recorded three times.

### 4.6. Lipidomic Analysis of AH in EAU

To prepare samples for mass spectrometric (lipidomic) analysis, AH was diluted with ice-cold anhydrous methanol, mixed with solutions of deuterated internal standards (2 ng) and centrifuged (12,000× *g*, 3 min). The supernatant was mixed with 0.1% acetic acid (6 mL) and loaded into a solid-phase lipid extractor (Oasis^®^PRIME HLB cartridge, Waters, Eschborn, Germany) (60 mg, 3 cc). The cartridge was washed with 2 mL of a 15% aqueous methanol solution containing 0.1% formic acid, and the lipids were sequentially eluted with 500 μL of anhydrous methanol and 500 μL of acetonitrile. The resulting samples were mixed and concentrated under a light stream of nitrogen. Signaling lipids of AH were analyzed on an 8040 series UPLC-MS/MS mass spectrometer (Shimadzu, Kyoto, Japan) in the multiple reaction monitoring mode with unit mass resolution for the precursor and product ions as described previously [83]. Lipids were separated by reversed-phase HPLC on a Phenomenex C8 column (0.4 mL/min, sample temperature 5 °C). Molecular ions were fragmented by collision-induced dissociation in the gas phase and analyzed by tandem (MS/MS) mass spectrometry. The isolated lipids were identified and quantified by comparing their mass spectrometric and chromatographic data with those obtained for the corresponding standards using a commercially available software method package (Lipid Mediator Version 2, Shimadzu, Kyoto, Japan).

### 4.7. Metablomic Analysis of AH in EAU

Twenty-five microliters of AH were mixed with 250 μL of ice-cold methanol, and after vigorous vortex stirring, the solution was centrifuged at 12,000× *g* at 4 °C for 5 min. The supernatant was dried under a light stream of nitrogen and stored at −80 °C. For NMR experiments, the dried extracts were dissolved in 500 µL of deuterated phosphate buffer (150 mM, pH 7.15) containing sodium azide (2 mM) as a preservative against bacteria and DSS-d6 (0.1 mM) as an internal standard. After centrifugation (10 min, room temperature), the samples were placed into standard 5 mm NMR tubes. The spectra were acquired on the AVANCE Neo 700 MHz NMR spectrometer (Bruker BioSpin, Reinstetten, Germany) equipped with a Prodigy triple resonance cryoprobe (Bruker BioSpin, Reinstetten, Germany) according to the parameters pointed out by Ivanova et al. [84]. The spectra were acquired at 25 °C using the noesypr1d pulse sequence with the following parameters: 131,072 data points, 4 dummy scans, 800 scans, 19.8364 ppm spectral width, with a 4.7 s acquisition time and 3.0 s relaxation delay between scans. Spectra processing and metabolite identification were performed in the Chenomx v9 (Chenomx Inc., Edmonton, AB, Canada) program based on the built-in database. The concentrations of metabolites were measured related to the DSS peak area in Chenomx v9 (Chenomx Inc., Edmonton, AB, Canada).

### 4.8. Statistical Analysis

Error bars in the figures represent mean ± SEM. Statistical significance was assessed using repeated measure ANOVA for analysis of the data obtained within the same group of animals before (control samples) and at different stages after (experimental samples) immunization) and one-way ANOVA (for analysis of the data obtained from groups of animals with and without the treatment), followed by pairwise comparisons using Tukey’s test. Probability less than 0.05 was considered significant. Statistical analysis and plotting were performed using the SigmaPlot 15.0 program (Systat Software, San Jose, CA, USA).

## 5. Conclusions

In this study, we proposed a novel rabbit model of posterior EAU induced by immunization with photoreceptor protein recoverin, which shows several benefits over previously developed models, including more rapid development and accurate reproduction of human disease. The model allowed us to comprehensively characterize the pathological process using clinical, electrophysiological and histological approaches, as well as biochemical and omics methods. Our findings suggest a number of potential therapeutic options for the disease, including the blockade of TNF-α/IL-6 and PAF signaling, inhibition of prostaglandin synthesis by COX-2 (non-steroidal anti-inflammatory drugs), DHA and ascorbic acid supplementation and/or antioxidant therapy. The new EAU model can be used to test single or combined versions of these or other treatments for posterior uveitis, as has been successfully demonstrated with therapy using the mitochondria-targeted antioxidant SkQ1.

## Data Availability

The data obtained in this study are available upon reasonable request from the corresponding author.
